# Common variants in *FKBP5* gene and major depressive disorder (MDD) susceptibility: a comprehensive meta-analysis

**DOI:** 10.1038/srep32687

**Published:** 2016-09-07

**Authors:** Shuquan Rao, Yao Yao, Joanne Ryan, Tao Li, Duan Wang, Chuan Zheng, Yong Xu, Qi Xu

**Affiliations:** 1 School of Life Science and Engineering, Southwest Jiaotong University, Chengdu 610031, China; 2 Department of Fundamental Medicine, Chengdu University of Traditional Chinese Medicine, Chengdu 611137, China; 3 Disease Epigenetics Group, Murdoch Childrens Research Institute & Department of Paediatrics, University of Melbourne, 3052 Parkville, Victoria, Australia; 4 Inserm, U1061, Univ Montpellier, F-34093 Montpellier, France; 5 Mental Health Center, West China Hospital/West China School of Medicine, Sichuan University, Chengdu, 610041, China; 6 Department of Orthopedics, West China Hospital/West China School of Medicine, Sichuan University, Chengdu, 610041, China; 7 Department of Psychiatry, First Clinical Medical College/First Hospital of Shanxi Medical University, Taiyuan, 030000, China; 8 National Laboratory of Medical Molecular Biology, Institute of Basic Medical Sciences & Neuroscience Center, Chinese Academy of Medical Sciences and Peking Union Medical College, Beijing 10005, China

## Abstract

Previous studies have investigated the association between common variants in *FKBP5* and MDD; however, the results remain inconsistent. In order to conduct a comprehensive meta-analysis of the association between *FKBP5* variants and MDD risk, seven studies involving 26582 subjects, including 12491 cases with MDD and 14091 controls, were enrolled totally. Four common SNPs (rs1360780, rs4713916, rs3800373 and rs755658) with complete data from two or more studies were analyzed. In the total sample, there was no evidence of a significant association between MDD and any of the four SNPs using a random-effects model. However, after removing one heterogeneous German study, as indicated by sensitivity analysis, both the rs1360780 T-allele (Z = 2.95, *P* = 0.003, OR = 1.06, 95% CI = 1.02–1.11) and the rs3800373 C-allele (Z = 3.05, *P* = 0.002, OR = 1.07, 95% CI 1.02–1.12) were significantly associated with MDD in a fixed-effect model. Our study thus provides support for an association between specific *FKBP5* genetic variants and MDD risk. Rs4713916 was not significantly associated with MDD; However, this analysis had limited statistical power and larger sample sizes are required to further validate this result. Future research should also investigate possible gender- and ethnicity-specific differences in the association between FKBP5 and MDD.

Major depressive disorder (MDD) is one of the most common psychiatric disorders, affecting up to 20% of the world population across their lifetime^1^. Some of the core symptoms of MDD include persistent depressed mood, loss of interest or pleasure, and psychomotor retardation. The World Health Organization has predicted that MDD will be the second leading cause of disability worldwide by the year 2020^2^.

Though the pathogenesis of MDD remains largely unknown, genetic factors have been shown to play an important role in conferring vulnerability to MDD and the heritability of MDD, estimated by family, twin and adoption studies, is 35–40%^3^,^4^. Uncovering the genetic basis of susceptibility to MDD has become an important task. Though risk variants range widely in frequency and penetrance^5^, together common variants identified by candidate gene or genome-wide association studies (GWAS) constitute a considerable portion of the genetic architecture of MDD^6^,^7^.

As opposed to GWASs, candidate gene studies investigate MDD genetic risk factors based on hypothesis driven approaches. Candidate genes of MDD have logically focused on the stress hormone-regulating hypothalamic-pituitary-adrenal (HPA) axis^8^ and neurotransmitter signaling pathways^9^, given their involvement in the development of MDD. Up till now, several dozen MDD susceptibility genes have been identified through candidate gene studies, for example, ACSM1^10^ and BDNF^11^.

FKBP5 is located on chromosome 6p21.31 (GRCh38), a region associated with psychosis^12^, and encodes the heat shock protein 90 co-chaperone. On one hand, the expression of FKBP5 is regulated by corticosteroids at the transcriptional level through the hormone response element (HRE). On the other hand, FKBP5 can decrease the affinity of the glucocorticoid receptor (GR) to corticosteroids by a complex interaction with the mature GR, thus resulting in impaired GR signaling^13^,^14^. Given the regulatory role of FKBP5 in HPA axis signaling, Binder *et al.* first investigated the association between common variants in *FKBP5* and MDD risk. They found that single nucleotide polymorphisms (SNPs) were significantly associated with the recurrence of depressive episodes and response to antidepressants, although no significant association was observed between SNPs and MDD^15^. Following this study, several other groups have reported an association between FKBP5 variants and MDD using American, German and Polish cohorts respectively^16^,^17^,^18^. However, other studies involving Spanish, Swedish and Italian populations have failed to replicate these finding^19^,^20^,^21^. Whether common variants in FKBP5 are associated with MDD susceptibility thus remains inconclusive.

Possible explanations for these inconsistent results might be genetic heterogeneity of MDD and allelic differences between ethnicities. Furthermore, the sample sizes in previous studies were relatively small and might be underpowered to detect the likely small effect sizes. Meta-analysis allows one to combine data from different studies, thus maximizing the statistical power to detect an association between common variants and MDD risk, if indeed one exists^22^. In this study, we report a comprehensive meta-analysis of the association between common *FKBP5* variants and MDD risk.

## Results

### Eligible studies

**Figure 1:**
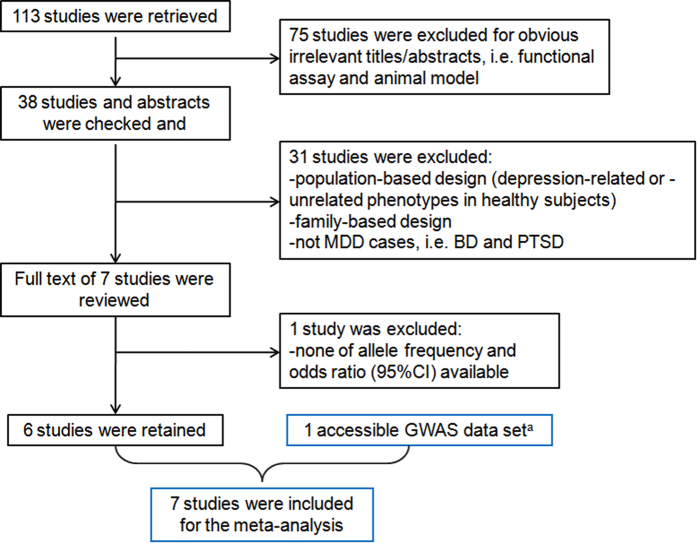
Literature search flow chart. ^a^The association result is accessible from Ricopili (http://www.broadinstitute.org/mpg/ricopili/). BD, bipolar disorder; PTSD, posttraumatic stress disorder.

According to the literature search strategy, a total of 113 references were identified. We excluded 107 studies for reasons specified in Fig. 1, leaving 6 independent studies for the meta-analysis^16^,^17^,^18^,^19^,^20^,^21^.

Although Binder *et al.* genotyped 5 SNPs across the *FKBP5* locus in a German cohort (294 MDD cases and 338 controls), and reported no evidence of an association with MDD, none of the exact genotype frequency data in the case and control groups was available, either from the manuscript^15^ or by contacting the corresponding author. Besides, the samples used in the Binder *et al.* study were also part of the European GWAS sample. Therefore, we excluded this sample from this meta-analysis. One European GWAS data set (9240 cases with MDD and 9519 controls), which reported the *P*-values, ORs and S.Es., was included in our meta-analysis^23^. Overall, 7 studies totaling 12491 cases with MDD and 14091 controls were combined in the present study. Careful examination of the details of each sample, ensured there was no overlap between the population included in the six association studies and the GWAS. Table 1 lists the information on the recruited studies, including sample size, ethnicity, mean age, gender ratio, diagnosis of MDD, and genotyping method.

To date, 11 SNPs in FKBP5 have been investigated in candidate gene association studies of MDD. Seven of these SNPs were reported in only one study and thus excluded from the meta-analysis (Table S1).The remaining 4 SNPs were rs1360780, rs4713916, rs3800373 and rs755658 (Table 2).

### Assessment of publication bias

Stata12.0 software was used to detect potential publication bias in the meta-analysis. No evidence of publication bias was observed for any of the SNPs: rs1360780 (Begg’s *P* = 0.652, Egger’s *P* = 0.862); rs4713916 (Begg’s *P* = 0.117, Egger’s *P* = 0.645); *rs3800373* (Begg’s *P* = 0.174, Egger’s *P* = 0.581) (funnel plots not shown), and rs755668 (Begg’s *P* = 0.117, Egger’s *P* = 0.669).

### Power analysis

Before the pooling procedure, statistical power was assessed with the following assumptions: *P* = 0.05, OR = 1.20 (corresponding to a “weak to moderate” gene effect) for the four SNPs, and minor allele frequencies (MAF) were estimated from the 1000 Genomes project (www.1000genomes.org/). The present sample size indicated 100% power for rs1360780 (MAF = 0.327) and rs3800373 (MAF = 0.325), 88% power for rs755658 (MAF = 0.059), and 57.9% power for rs4713916 (MAF = 0.222, 2013 cases and 1274 controls available) to detect a significant association with MDD, if one indeed existed.

### FKBP5 variants and MDD susceptibility

**Figure 2:**
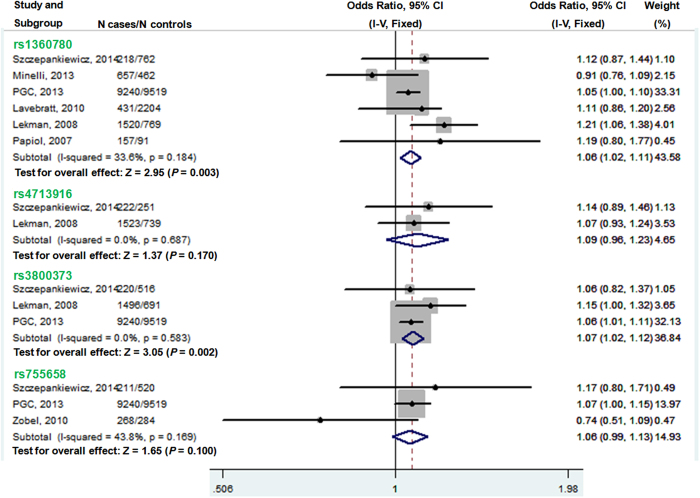
Forest plot of meta-analysis for rs1360780 T-allele, rs4713916 A-allele, rs3800373 C-allele, and rs755668 T-allele of *FKBP5* gene using fixed-effect model. Note: I-V, Inverse-variance; PGC, Psychiatric GWAS Consortium.

There was significant evidence of between-study heterogeneity for the rs1360780 T-allele (I^2^ = 56.7%, *P* = 0.031), rs4713916 A-allele (I^2^ = 75.5%, *P* = 0.017) and rs3800373 C-allele (I^2^ = 68.4%, *P* = 0.023), therefore the random-effects model was used to combine samples. Meta-analysis of all available samples showed that none of the three SNPs, rs1360780 (OR = 1.05, 95% CI 0.95–1.15, Z = 0.97, *P* = 0.333), rs4713916 (OR = 0.97, 95% CI 0.76–1.24, Z = 0.24, *P* = 0.812) and rs3800373 (OR = 1.02, 95% CI 0.89–1.17, Z = 0.28, *P* = 0.779), was associated with MDD (Fig. S1). Furthermore, there was no association between the rs755658 T-allele and MDD with the fixed-effect model (OR = 1.06, 95% CI 0.99–1.13, Z = 1.65, *P* = 0.100), since no significant heterogeneity was observed among the different studies for this SNP (I^2^ = 43.8%, *P* = 0.169) (Fig. 2).

### Sensitivity analyses

Considering the significant heterogeneity observed for rs1360780 T-allele, rs4713916 A-allele and rs3800373 C-allele, we conducted sensitivity analysis in order to determine the source of heterogeneity which might influence the findings. The sensitivity analysis indicated a large difference in the heterogeneity of the population for rs1360780 (from I^2^ = 56.7%, *P* = 0.031, to I^2^ = 33.6%, *P* = 0.184), rs4713916 (from I^2^ = 75.5%, *P* = 0.017, to I^2^ = 0%, *P* = 0.687), as well as rs3800373 (from I^2^ = 68.4%, *P* = 0.023, to I^2^ = 0.0%, *P* = 0.687), after removing the Zobel *et al.* study^17^.

A careful examination of the Zobel *et al.* study showed that 1) both the rs1360780 T-allele and the rs4713916 A-allele from the German population had the highest MAF and protective association with MDD, whereas the other studies indicated that both the other two SNPs contributed to MDD risk (Table 2); and 2) MAF of rs1360780 (0.339) was considerably different from the HapMap project (MAF = 0.268 in European samples).

After removing the Zobel *et al.* study, we pooled ORs again with the fixed-effect model. Meta-analysis showed that the rs1360780 T-allele was significantly associated with MDD (Z = 2.95, *P* = 0.003) with OR 1.062 (95% CI 1.02–1.11). For rs3800373, the C-allele conferred significantly increased MDD risk to MDD as well (Z = 3.05, *P* = 0.002) with an OR 1.07 (95% CI 1.02–1.12). However, there remained no significant association between MDD and rs4713916 (Z = 1.37, *P* = 0.170, OR = 1.09, 95% CI 0.96–1.23) (Fig. 2).

## Discussion

It has been recognized as one of the greatest challenges to decipher the genetic architecture underlying MDD. Most of the inconsistent association results of MDD might be due to limited sample sizes, likely lacking statistical power, and heterogeneous patient populations, i.e. ethnicity and subtypes of MDD. Meta-analysis is a widely accepted technique used to produce solid conclusions by combining data from independent studies together^24^, and more importantly, serve as a powerful method to dissect potential source of heterogeneity. In the present study, we conducted a comprehensive meta-analysis of common variants across the *FKBP5* locus and MDD risk. The meta-analysis involved a total sample size of 26582, including 12491 patients and 14091 healthy controls, which was sufficiently powered to detect MDD risk associated with genetic factors that had a low to moderate effect size (OR = 1.20). Although we found no significant evidence of an association between any of the SNPs in *FKBP5* and MDD susceptibility when all samples were pooled, when one heterogeneous study was removed^17^, both the rs1360780 T-allele (Z = 2.95, *P* = 0.003, OR = 1.062, 95% CI 1.02–1.11) and the rs3800373 C-allele (Z = 3.05, *P* = 0.002, OR = 1.07, 95% CI 1.02–1.12) were significantly associated with MDD (Fig. 2).

No prior individual study has reported a significant association of MDD with either rs1360780 or rs3800373. For rs1360780, the majority of studies found that the T-allele frequency was higher among cases than in controls, with a moderate effect (OR ranging from 1.049 to1.210)^16^,^18^,^19^,^20^,^23^ but failed to reach statistical significance. Two studies have reported small effect sizes in the reverse direction (OR ranging from 0.762 to 0.912)^17^,^21^. For rs3800373, Lekman *et al.* reported a marginal association with MDD (P = 0.052)^17^. As we know, the statistical power of a case-control genetic association study is determined by a number of factors including the type I error probability, OR, the MAF of the genetic variants and the sample size. Simplified, the required sample size increases exponentially as the ORs declines. To detect a SNP with an OR of 1.1 or less, tens of thousands of samples will be required (based on 10% prevalence of MDD)^25^. Given the observed small effect size of the rs1360780 T-allele and the rs3800373 C-allele shown in this meta-analysis, this suggests that previous studies of this variant were under-powered, thus providing a possible explanation for their null findings^26^.

Differences in the allelic frequencies of a given SNP across ethnic groups, can influence the findings^27^. Given that the studies included in the meta-analysis involve predominantly individuals of European ancestry, it’s difficult to generalize these findings to other ethnic groups. For example, SNP rs1344707 in ZNF804A is significantly associated with schizophrenia risk in European populations, however is not in a Chinese population^28^. As is shown in the HapMap project (http://hapmap.ncbi.nlm.nih.gov/), the T-allele frequencies of rs1360780 in Japanese and Nigerian were 0.210 and 0.449 respectively, being quite different from that in European samples (0.268). Whether the rs1360780 T-allele or the rs3800373 C-allele are associated with MDD in non-European populations (i.e., Asian and African) remains to be determined. Furthermore, a number of genetic variants confer risk to MDD in a gender-specific way. For example, a meta-analysis of BDNF Val66Met polymorphism found that this variant was not significantly associated with MDD in the overall population or among females, but was associated with MDD in males (OR = 1.27, 95% CI 1.10–1.47)^11^. The rs619002 and rs644926 SNPs of the EHD3 locus were exclusively associated with MDD in females but not in males^29^. Given that each of the studies included in the meta-analysis did not provide genotypic information separately for each gender, the potential gender-specific association between rs1360780 T-allele and MDD was not analyzed. Whether or not the other FKBP5 SNPs, especially rs4713916 and rs755658, were associated with MDD in a gender-specific way also remains unknown. In addition, although our meta-analysis have the distinct advantage of combining data from multiple studies, strengthening the overall findings, the estimated statistical power to investigate the association between rs4713916 and MDD was indeed limited (57.9%), which might help explain the null finding. It remains possible that this SNP is associated with MDD, but this can only be association may be identified in even larger sample sizes.

FKBP5 is a heat shock protein 90 co-chaperone, which is essential for maturation and activation of GR^13^. In the absence of corticosteroids, FKBP5 can decrease the affinity of GR to corticosteroids by a complex interaction with the mature GR, thus leading to weakened GR signaling^30^. There is evidence to suggest that rs1360780 TT homozygous carriers have higher FKBP5 levels, and these are twice as high as those with C+ genotypes (*P* = 0.024)^15^. These different expression patterns of FKBP5 might be due to different transcriptional activity of FKBP5, which is regulated by a conserved HRE located less than 200 bp away from rs1360780 in intron 2^31^. This FKBP5 variant has also been associated with hippocampal volume, an endophenotype of MDD^17^. Moreover, rs1360780 could regulate the sensitivity of GR to its ligand^15^. Furthermore work should now focus on exploring the functional differences of rs1360780 alleles, which would provide further insights into the involvement of this gene in MDD.

## Methods

### Search strategy

Meta-analysis was performed in agreement with previously described methods^24^,^32^. To identify eligible studies for the meta-analysis, PubMed (http://www.ncbi.nlm.nih.gov), SCOPUS (http://www.scopus.com), EMBASE (http://www.elsevier.com/online-tools/embase) and ISI Web of Knowledge (http://apps.webofknowledge.com/) were retrieved with the following searching terms “(FKBP5 or FKBP51 or FKBP54) and (depression or mood disorder or affective disorder)”. Since FKBP5 is also known as FKBP51 or FKBP54, the three gene symbols were retrieved simultaneously. Studies published in English before the 1st of November 2015 were considered. The references of retrieved articles were also reviewed to identify other eligible studies that were not indexed by the above-mentioned databases.

### Inclusion and exclusion criteria

Only those studies investigating at least one FKBP5 SNP were included in the current meta-analyses. Eligible studies were also required to meet the following criteria: 1) be published in a peer-reviewed journal; 2) a case-control studies; 3) provide genotype and/or allele frequencies in both case and control samples, or statistics, including odds ratio (OR) with 95% confidence interval (95% CI); 4) samples were independent of other studies (if samples from different studies overlapped, only the first published data was included in this meta-analysis); 5) MDD was assessed in accordance with DSM-III, DSM-IIIR, DSM-IV, DSM-V (Diagnostic and Statistical Manual of Mental Disorders) or ICD-10 (International Classification of Diseases) diagnostic criteria; and 6) genotype frequencies in the controls were in Hardy–Weinberg equilibrium (HWE) (*P* > 0.05).

Studies with any of the following criteria were excluded from this meta-analysis: 1) case-only studies, family-based designs and population studies with healthy subjects which were designed to explore the effects of FKBP5 variants on depression-related phenotypes, i.e., anxiety or personality traits; 2) studies with insufficient data to calculate an effect size, even after we contacted the corresponding authors.

Eligible studies included a European GWAS data set, which was freely accessible from Ricopili (http://www.broadinstitute.org/mpg/ricopili/)^23^. We extracted information on FKBP5 SNPs, including *P*-value, odds ratio (OR) and 95% confidence intervals (95% CI) and standard error (S.E.). This GWAS enrolled a total of 9240 cases with MDD and 9519 controls, from nine distinct groups, including GAIN (Genetic Association Information Network studies, 1696 cases and 1765 controls), GenRED (Genetics of Recurrent Early-onset Depression, 1030 cases and 1253 controls), GSK (Glaxo-Smith-Kline, 887 cases and 864 controls), MDD2000-QIMR_610 (433 cases and 751 controls), MDD2000-QIMR_317 (1017 cases and 960 controls), MPIP (376 cases and 537 controls), RADIANT + Bonn/Mannhein (935 cases and 1290 controls), RADIANT (1625 cases and 1588 controls), and STAR*D (Sequenced Treatment Alternatives to Relieve Depression, 1241 cases and 511 controls)^6^,^33^,^34^,^35^,^36^,^37^,^38^,^39^.

### Data extraction

For each eligible study, two independent investigators (Rao and Yao) extracted the following data using a standardized data extraction form: 1) first author and publication year; 2) study design; 3) sample origin; 4) sample size, gender ratio and age distribution if available; 5) MDD diagnosis criteria; 6) OR and 95% CI; and 7) genotypic and allelic distribution of cases and controls. If essential data were not available directly from the manuscripts, we calculated these values using the existing data, or contacted the authors for additional data.

### Statistical analysis

Chi-square (X^2^) goodness-of-fit test was used to calculate the Hardy Weinberg Equilibrium (HWE) of genotype frequencies in controls, when this was not reported. *P* < 0.05 was used as the threshold of statistical significance. We applied the Power and Sample Size Program software to perform power analysis^40^, and Stata12.0 statistical software package (http://www.stata.com/) to conduct publication bias analysis, meta-analysis and sensitivity analysis. Potential publication bias was checked using the Egger regression test for a funnel plot^41^ and the Begg–Mazumdar test, which is based on Kendall’s-τ^42^.

Cochran’s x^2^-based Q-statistic was performed to assess the heterogeneity between individual OR estimates. The extent of inconsistency across studies was quantified with the I^2^ metric (I^2^ = Q-d.f./Q) that takes values between 0 and 100%, with 0–25% representing no heterogeneity, 25–50% moderate heterogeneity, 50–75% large heterogeneity and 75–100% extreme heterogeneity^43^. When heterogeneity was present, a random-effects model was used to combine the odds ratio and the corresponding 95% CI; otherwise, a fixed-effect model was used. The significance of the pooled OR was determined by the Z test. Sensitivity analysis was performed to test the potential effect of individual studies on the pooled OR by sequentially removing each study and recalculating the pooled OR and 95% CI. For all analyses, *P* < 0.05 was considered statistically significant.

## Additional Information

**How to cite this article**: Rao, S. *et al.* Common variants in *FKBP5* gene and major depressive disorder (MDD) susceptibility: a comprehensive meta-analysis. *Sci. Rep.* **6**, 32687; doi: 10.1038/srep32687 (2016).

## Supplementary Material

Supplementary Information

## Figures and Tables

**Table 1:** Characteristics of included studies and samples on the association of *FKBP5* variants and MDD.

Author, Year	Ethnicity	MDD cases			Healthy controls			Definition of MDD	Genotyping method
		N^a^	Mean age	Gender (% Male)	N^a^	Mean age	Gender (% Male)		
Szczepankiewicz, 2014	Poland	218	45.5 ± 14.0	50 (23.0)	528	37.7 ± 12.5	315 (42.5)	DSM-IV	TaqMan
Minelli 2013	Italy	657	56.1 ± 13.7	210 (32.0)	462	46.7 ± 16.3	203 (43.9)	DSM-IV	Illumina
Zobel 2010	Germany	268	48.9 ± 14.0	98 (36.6)	284	46.5 ± 15.0	115 (40.5)	DSM-IV	TaqMan
Lavebratt 2010	Sweden	431	44.9 ± 12.0	118 (27.3)	2204	45.1 ± 12.1	968 (43.9)	DSM-IV	TaqMan
Lekman 2008	USA	1520	N.A.	N.A.	769	N.A.	N.A.	DSM-IV	Illumina, TaqMan
Papiol 2007	Spain	159	39.5 ± 12.2	35 (22.0)	96	35.1 ± 10.3	55 (57.3)	DSM-IV	TaqMan
Psychiatric GWAS Consortium, 2013	European^b^	9240	*See below	*See below	9519	*See below	*See below	DSM-IV	Illumina, Affymetrix

^a^The overall sample recruited in each study.

^b^samples from 9 distinct populations of European ancestry.

^*^This study was a GWAS mega-analysis of MDD and the age and gender information was available in each study, respectively.

N.A., not available.

**Table 2:** Characteristics of the association studies between 4 SNPs of *FKBP5* included for meta-analysis and MDD.

SNP (Major/minor allele)	Position	Author, year	Ethinicity	aN cases/N controls	Minor allele frequency		*P*-value	OR (95% CI)
					Cases	Controls		
rs1360780 (C/T)	Chr6: 35639794	Szczepankiewicz, 2014	Poland	218/762	0.280	0.257	0.372	1.121 (0.872–1.442)
		Minelli, 2013	Italy	657/462	0.312	0.332	0.313	0.912 (0.761–1.091)
		Zobel, 2010	Germany	268/284	0.281	0.339	0.036	0.762 (0.590–0.984)
		Lavebratt, 2010	Sweden	431/2204	0.268	0.265	0.844	1.107 (0.862–1.199)
		Lekman, 2008	USA	1520/769	0.346	0.304	0.005	1.210 (1.060–1.380)
		Papiol, 2007	Spain	157/91	0.334	0.297	0.386	1.191 (0.802–1.768)
		Psychiatric GWAS Consortium, 2013	bEuropean	9240/9519	*See below	*See below	0.039	1.049 (1.002–1.098)
rs755658 (C/T)	Chr6: 35581893	Szczepankiewicz, 2014	Poland	211/520	0.104	0.090	0.411	1.171 (0.803–1.708)
		Zobel, 2010	Germany	268/284	0.094	0.122	0.126	0.744 (0.506–1.093)
		Psychiatric GWAS Consortium, 2013	European	9240/9519	*See below	*See below	0.067	1.068 (0.995–1.146)
rs4713916 (G/A)	Chr6: 35702206	Szczepankiewicz, 2014	Poland	222/521	0.284	0.258	0.306	1.139 (0.888–1.460)
		Zobel, 2010	Germany	268/284	0.273	0.341	0.014	0.722 (0.558–0.934)
		Lekman, 2008	USA	1523/739	0.275	0.261	0.322	1.074 (0.933–1.236)
rs3800373 (A/C)	Chr6: 35574699	Szczepankiewicz, 2014	Poland	220/516	0.255	0.244	0.673	1.057 (0.817–1.367)
		Zobel, 2010	Germany	268/284	0.255	0.322	0.014	0.722 (0.556–0.939)
		Lekman, 2008	USA	1496/691	0.328	0.298	0.052	1.147 (0.999–1.317)
		Psychiatric GWAS Consortium, 2013	European	9240/9519	*See below	*See below	0.011	1.062 (1.014–1.113)

^a^The N represents the number of individuals having genotyping data.

^b^samples from 9 distinct populations of European ancestry.

^*^Only the MAF of the overall samples available (rs1360780: 0.308; rs755668: 0.067; rs3800373: 0.291).
