# Supplementary material for: Common variants in FKBP5 gene and major depressive disorder (MDD) susceptibility: a comprehensive meta-analysis
Source: Sci Rep. 2016 Sep 7;6:32687. doi: 10.1038/srep32687 (PMC5013409; doi:10.1038/srep32687)
Supplement: Supplementary Information [file srep32687-s1.doc]

This document included the supplementary materials for:

**Common variants in *FKBP5* gene and major depressive disorder (MDD) susceptibility: a comprehensive meta-analysis**

Shuquan Rao^1,9,＊^, Yao Yao^2,9^, Joanne Ryan^3,4,9^, Tao Li^5^, Duan Wang^6^, Chuan Zheng^2^,

Yong Xu^7^, Qi Xu^8,＊^

^1^School of Life Science and Engineering, Southwest Jiaotong University, Chengdu 610031, China

^2^Department of Fundamental Medicine, Chengdu University of Traditional Chinese Medicine, Chengdu 611137, China

^3^Disease Epigenetics Group, Murdoch Childrens Research Institute & Department of Paediatrics, University of Melbourne, 3052 Parkville, Victoria, Australia

^4^Inserm, U1061, Univ Montpellier, F-34093 Montpellier, France

^5^Mental Health Center, West China Hospital/West China School of Medicine, Sichuan University, Chengdu, 610041, China

^6^Department of Orthopedics, West China Hospital/West China School of Medicine, Sichuan University, Chengdu, 610041, China

^7^Department of Psychiatry, First Clinical Medical College/First Hospital of Shanxi Medical University, Taiyuan, 030000, China

^8^National Laboratory of Medical Molecular Biology, Institute of Basic Medical Sciences & Neuroscience Center, Chinese Academy of Medical Sciences and Peking Union Medical College, Beijing 10005, China

^9^These authors contributed equally to this work.

Correspondence and requests for materials should be addressed to S.R. (email: [shuquan_rao@163.com](mailto:shuquan_rao@163.com)) or Q.X. (email: xuqi@pumc.edu.cn).

Content

[Supplementary table 1 4](#__RefHeading___Toc437334868)

[Supplementary figure 1 5](#__RefHeading___Toc437334869)

**Supplementary table 1: Characteristics of the association studies between 7 SNPs of *FKBP5* excluded for this meta-analysis**

| SNP (minor allele/Major allele) | Position | Author, year | Ethnicity | *N* cases/*N* controls^a^ | Minor allele frequency | | *P*-value | OR (95%CI) |
| --- | --- | --- | --- | --- | --- | --- | --- | --- |
|  |  |  |  |  | Cases | Controls |  |  |
| rs9470080 | Chr6: 35678658 | Szczepankiewicz, 2014[^1^](#_ENREF_1) | Poland | 217/516 | 0.311 | 0.295 | 0.529 | 1.081 (0.848-1.379) |
| rs7748266 | Chr6: 35624967 | Szczepankiewicz, 2014[^1^](#_ENREF_1) | Poland | 222/523 | 0.142 | 0.131 | 0.572 | 1.097 (0.796-1.513) |
| rs9296158 | Chr6: 35599305 | Szczepankiewicz, 2014[^1^](#_ENREF_1) | Poland | 211/516 | 0.277 | 0.258 | 0.444 | 1.105 (0.856-1.425) |
| rs9394309 | Chr6: 35654004 | Szczepankiewicz, 2014[^1^](#_ENREF_1) | Poland | 215/515 | 0.286 | 0.272 | 0.580 | 1.073 (0.836-1.378) |
| rs7757037 | Chr6: 35580459 | [Minelli](http://www.ncbi.nlm.nih.gov.pbidi.unam.mx:8080/pubmed/?term=Minelli A%5BAuthor%5D&cauthor=true&cauthor_uid=23861224), 2013[^2^](#_ENREF_2) | Italy | 653/461 | 0.508 | 0.475 | 0.121 | 1.143 (0.965-1.353) |
| rs4713902 | Chr6: 35646249 | [Minelli](http://www.ncbi.nlm.nih.gov.pbidi.unam.mx:8080/pubmed/?term=Minelli A%5BAuthor%5D&cauthor=true&cauthor_uid=23861224), 2013[^2^](#_ENREF_2) | Italy | 655/461 | 0.321 | 0.282 | 0.051 | 1.202 (0.999-1.445) |
| rs1334894 | Chr6: rs1334894 | Zobel, 2010[^3^](#_ENREF_3) | Germany | 268/284 | 0.09 | 0.123 | 0.070 | 0.700 (0.475-1.031) |

^a^The N represents the number of individuals having genotyping data.

**Supplementary figure 1** **Forest plot of meta-analysis for rs1360780 T-allele, rs4713916 A-allele, rs3800373 C-allele of *FKBP5* gene using random-effect model**


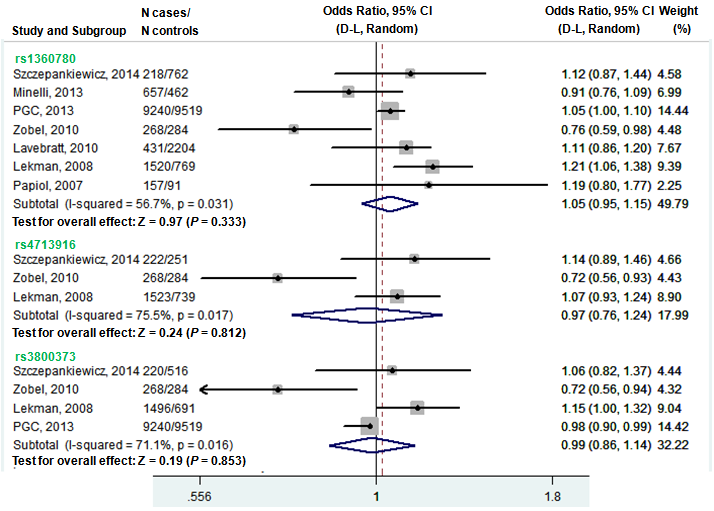


Note: D-L, DerSimonian-Laird; PGC, Psychiatric GWAS Consortium.

**Supplementary references**

1 Szczepankiewicz, A. *et al.* FKBP5 polymorphism is associated with major depression but not with bipolar disorder. *J Affect Disord* **164**, 33-37 (2014).

2 Minelli, A. *et al.* Role of allelic variants of FK506-binding protein 51 (FKBP5) gene in the development of anxiety disorders. *Depression and anxiety* **30**, 1170-1176 (2013).

3 Zobel, A. *et al.* DNA sequence variants of the FKBP5 gene are associated with unipolar depression. *The international journal of neuropsychopharmacology / official scientific journal of the Collegium Internationale Neuropsychopharmacologicum* **13**, 649-660 (2010).
